# Distinguishable *In Vitro* Binding Mode of Monomeric TRBP and Dimeric PACT with siRNA

**DOI:** 10.1371/journal.pone.0063434

**Published:** 2013-05-02

**Authors:** Tomoko Takahashi, Takuya Miyakawa, Shuhei Zenno, Kenji Nishi, Masaru Tanokura, Kumiko Ui-Tei

**Affiliations:** 1 Department of Biophysics and Biochemistry, Graduate School of Science, The University of Tokyo, Tokyo, Japan; 2 Department of Applied Biological Chemistry, Graduate school of Agricultural and Life Sciences, The University of Tokyo, Tokyo, Japan; 3 Department of Biotechnology, Faculty of Engineering, Maebashi Institute of Technology, Gunma, Japan; Griffith University, Australia

## Abstract

RNA interference (RNAi) is an evolutionally conserved posttranscriptional gene-silencing mechanism whereby small interfering RNA (siRNA) triggers sequence-specific cleavage of its cognate mRNA. Dicer, Argonaute (Ago), and either TAR-RNA binding protein (TRBP) or a protein activator of PKR (PACT) are the primary components of the RNAi pathway, and they comprise the core of a complex termed the RNA-induced silencing complex (RISC)-loading complex (RLC). TRBP and PACT share similar structural features including three dsRNA binding domains (dsRBDs), and a complex containing Dicer and either TRBP or PACT is considered to sense thermodynamic asymmetry of siRNA ends for guide strand selection. Thus, both TRBP and PACT are thought to participate in the RNAi pathway in an indistinguishable manner, but the differences in siRNA binding mode and the functional involvement of TRBP and PACT are poorly understood. Here, we show *in vitro* binding patterns of human TRBP and PACT to siRNA using electrophoresis mobility shift analysis and gel filtration chromatography. Our results clearly showed that TRBP and PACT have distinct *in vitro* siRNA binding patterns from each other. The results suggest that monomeric TRBP binds to siRNA at the higher affinity compared to the affinity for own homodimerization. In contrast, the affinity between PACT and siRNA is lower than that of homodimerization or that between TRBP and siRNA. Thus, siRNA may be more readily incorporated into RLC, interacting with TRBP (instead of PACT) *in vivo*.

## Introduction

RNA interference (RNAi) and related pathways are evolutionally conserved mechanisms whereby single-stranded guide RNA triggers sequence-specific cleavage or translational repression of its cognate mRNA through the RNA-induced silencing complex (RISC) [1]–[3]. Silencing is initiated by long double-stranded RNA (dsRNA) or hairpin-structured precursor-microRNA (pre-miRNA), which are processed by the RNaseIII enzyme Dicer to produce 21–23 nucleotide (nt) short interfering RNAs (siRNAs) or microRNAs (miRNAs), respectively [4]–[6]. These small RNAs are then loaded onto Argonaute2 (Ago2), the slicer of target mRNA [7], [8]. In human cells, both Dicer and the trans-activation response (TAR) RNA binding protein (TRBP) or the protein activator of protein kinase R (PKR) (PACT) are associated with RISC loading [8]–[10]. These proteins, along with Ago2, comprise the core of a complex referred to as the RISC-loading complex (RLC) [11]–[13]. In human core RLC, a heterodimer containing Dicer and either TRBP or PACT can sense the thermodynamic asymmetry of siRNA ends *in vitro* [10]. Dicer interacts with the less stable end of the duplex, and TRBP/PACT interacts with the more stable end. Within these heterodimeric complexes, siRNAs undergo significant repositioning following dicing, thereby biasing their orientation for guide strand selection according to the thermodynamic properties of the helix [10]. Thus, Dicer itself can sense this asymmetry, and functionality is activated upon association with either TRBP or PACT. Furthermore, TRBP and PACT are known to interact with each other and associate with Dicer to facilitate dsRNA cleavage [14], [15].

TRBP and PACT also share structural similarities, each containing three dsRNA binding domains (dsRBDs), namely, dsRBD1, dsRBD2, and dsRBD3. TRBP was first identified as a factor that binds to human immunodeficiency virus-1 (HIV-1) TAR RNA [16]. RNA binding analyses of the three TRBP dsRBDs revealed that the first and second dsRBDs (dsRBD1 and dsRBD2) bind TAR RNA [17] and siRNA [18], [19], respectively, while the third (dsRBD3) binds neither of these RNAs and interacts with Dicer [9], [20]. Furthermore, TRBP forms heterodimers with PKR via dsRBD1 and dsRBD2 without dsRBD3, and inhibits the function of PKR [21]. However, PACT was initially thought to be a PKR activator [22]. Either PACT dsRBD1 or dsRBD2 are required for strong binding to PKR, and dsRBD3 is necessary and sufficient to activate PKR. Thus, PACT and TRBP have opposite roles in PKR regulation, although their PKR binding patterns are very similar. Meanwhile, PACT also binds to Dicer through dsRBD3, and stimulates the cleavage of dsRNA to siRNA [14] or enhances the function of siRNA strand selection by Dicer [10] in a very similar manner as TRBP. Furthermore, both PACT and TRBP form homodimer by the interaction within each of dsRBD1 and dsRBD2 domains, or in an interchangeable manner [21]. However, the siRNA binding mode of PACT remains unclear.

To distinguish between TRBP and PACT in the RNA silencing process, we compared the fundamental characteristics of human TRBP and PACT for siRNA binding using electrophoresis mobility shift analysis (EMSA) and gel filtration chromatography *in vitro*. Here, we propose that TRBP and PACT have unique siRNA binding modes *in vitro*, although they have similar structural and functional features of RNA silencing.

## Results

### Monomeric TRBP binds siRNA at low concentrations

**Figure 1:**
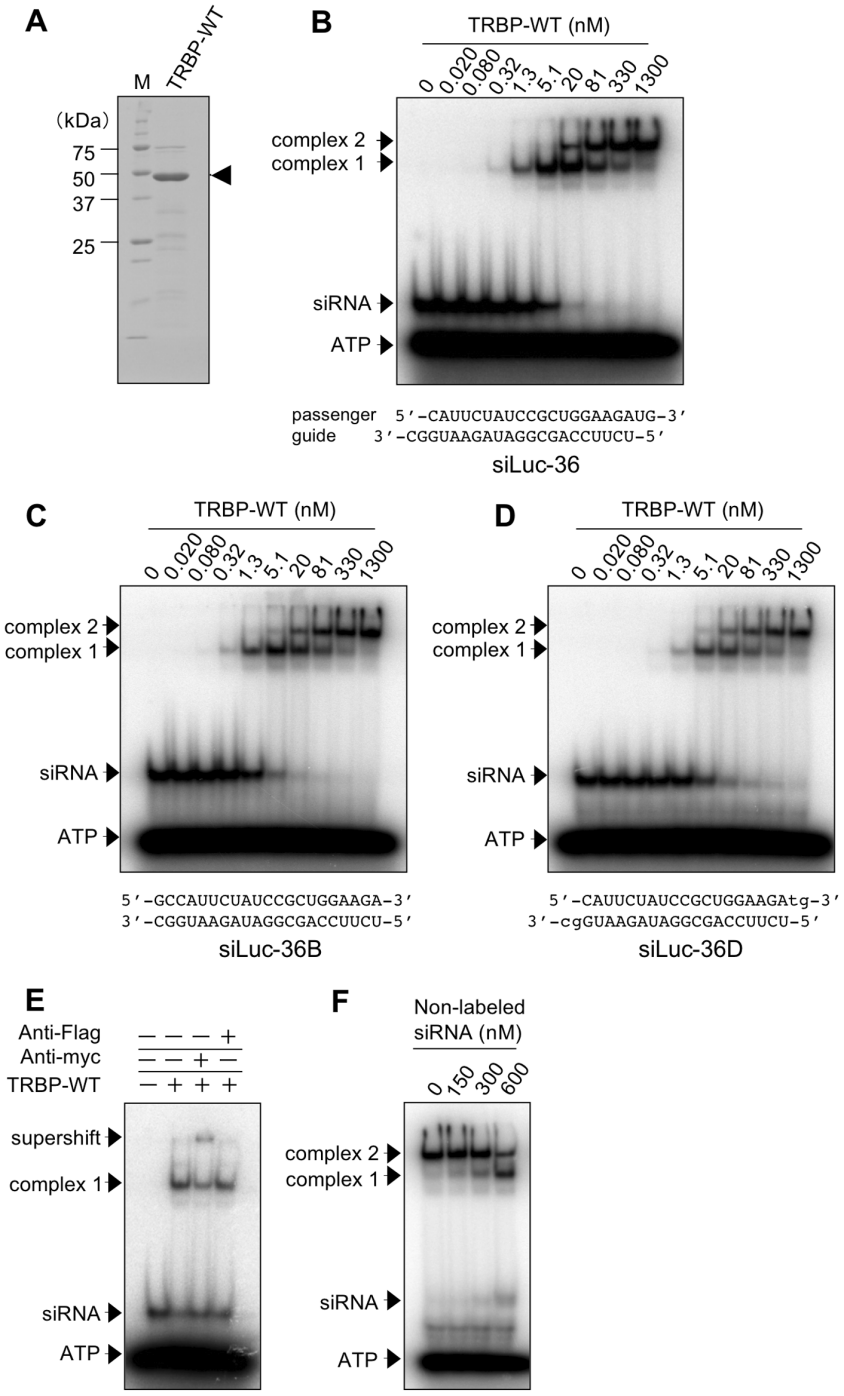
Purification and EMSA of TRBP-WT protein. (A) Coomassie brilliant blue stained pattern of purified TRBP-WT protein resolved by SDS-PAGE. Arrowhead indicates 44 kDa of TRBP-WT protein. M represents the protein size markers. (B–D) The results of EMSA of TRBP-WT protein with ^32^P-labeled siLuc-36 (B), siLuc-36B (C), and siLuc-36D (D). ^32^P-labeled siRNA (0.50 nM) was incubated with increasing amounts of TRBP-WT protein, as indicated. Lower cases in siLuc-36D sequence in D indicate DNAs. (E) Supershift analysis of TRBP-WT protein (1.3 nM) with no antibodies, control anti-Flag, and anti-myc antibodies. (F) The result of EMSA of TRBP-WT protein (1,300 nM) mixed with ^32^P-labeled siLuc-36 (0.50 nM) incubated with increasing amount of non-labeled siLuc-36. In B–F, arrows indicate positions of the first and second step migrating complexes, corresponding to TRBP-WT complexes 1 and 2, respectively, and the supershifted complex, in addition to siRNA and ATP.

To investigate the binding pattern of human TRBP protein to siRNA, a recombinant N-terminal His- and C-terminal myc-tagged wild-type TRBP protein (TRBP-WT) was expressed in *Escherichia coli* (*E. coli*) Rosetta (DE3) pLysS and purified on Ni-NTA agarose. This recombinant TRBP-WT protein was soluble and the homogeneity was evaluated by SDS-PAGE (Figure 1A). The binding ability of TRBP-WT protein to siRNA (0.50 nM) was examined by EMSA with 21 nt siRNA containing a ^32^P-labeled guide strand. The migrating ribonucleoprotein complexes composed of TRBP-WT protein and siRNA against firefly luciferase (siLuc-36) were obtained in two steps with TRBP-WT concentrations ranging from 0 to 1,300 nM (Figure 1B). We referred the complex in the first binding step as complex 1 and that in the second binding step as complex 2.

The same experiments were performed using the other five siRNAs against firefly luciferase (siLuc2-153, siLuc-30, siLuc-48, siLuc-18, and siLuc-1430) with different sequences (Figure S1A, C–F). In our previous reports, siLuc-36 and siLuc2-153 were categorized into highly functional siRNAs, siLuc-30 and siLuc-48 were siRNAs with intermediate degrees of silencing efficiencies, and siLuc-18 and siLuc-1430 were not functional with little or no silencing activities [23]. Regardless of their silencing efficiencies, all siRNAs showed similar binding patterns to the highly functional siRNAs, siLuc-36 (Figure 1B) and siLuc2-153 (Figure S1A). The siRNA-binding curve derived from the EMSA experiments performed at TRBP-WT concentrations of 0 to 1,300 nM revealed dissociation constants (*K*d) for the formation of complex 1 were ranged from 5.30 to 33.0 nM (mean 10.0 nM), and from 44.0 to 440 nM (mean 203 nM) for the formation of complex 2 (Table 1). These results indicate that TRBP-WT binding efficiencies to siRNAs are slightly varied in a sequence-dependent manner but not necessarily reflecting the silencing efficiencies. TRBP is shown to sense the thermodynamic asymmetry of siRNA duplex ends *in vitro* and interacts with more stable end of the duplex [24]. Then we examined the effects of 2 nt 3′ overhang nucleotides in both terminals using 21 nt dsRNAs with blunt ends (siLuc-36B and siLuc2-153B) and siRNAs containing DNAs at both 3′ overhangs (siLuc-36D and siLuc2-153D). The binding patterns of siLuc-36B and siLuc2-153B to TRBP-WT were similar to those of siLuc-36 and siLuc2-153 (Figures 1C and S1K) and the *K*_d_ values of siLuc-36B and siLuc2-153B calculated from siRNA-binding curves were 4.50 and 5.10 nM, respectively, for the formation of complex 1 (Table 1). siLuc-36D and siLuc2-153D also showed similar binding patterns to those of siLuc-36 and siLuc2-153 (Figures 1D and S1L) and their *K*_d_ values for the complex 1 formation were 11.0 and 5.50 nM, respectively (Table 1). The *K*d values of blunt-ended dsRNAs and siRNAs with DNAs at 3′ overhangs were close to the values of siLuc-36 and siLuc2-153 (5.30 and 4.00 nM, respectively), strongly suggesting that the nucleotides at 3′ overhangs does not affect the binding activity with TRBP, and TRBP mainly bind to a double stranded portion in siRNA. Consistent with these results, TRBP-WT protein did not bind the 21 nt double-stranded DNA (dsDNA) (Figure S1G and H) and 21 nt single-stranded RNA (Figure S1I and J).

**Table 1:** The *K*_d_ values (nM) of TRBP-WT and PACT-WT and their mutants.

Purified protein	complex	Highly functional		Intermediate		Not functional			Blunt-ended*			DNA at 3′ overhang**		
		siLuc- 36	siLuc2- 153	siLuc- 30	siLuc- 48	siLuc- 18	siLuc- 1430	Mean	siLuc- 36B	siLuc2- 153B	Mean	siLuc- 36D	siLuc2- 153D	Mean
**TRBP-WT**	**complex 1**	5.30	4.00	6.20	33.0	5.80	5.70	**10.0**	4.50	5.10	**4.80**	11.0	5.50	**8.25**
	**complex 2**	44.0	440	81.0	430	93.0	130	**203**	60.0	74.0	**67.0**	243	133	**188**
**TRBP- dsRBDmt1**	**complex**	250	260	NT	NT	NT	NT	**255**	NT	NT	**NT**	NT	NT	**NT**
**TRBP- dsRBDmt2**	**complex**	370	520	NT	NT	NT	NT	**445**	NT	NT	**NT**	NT	NT	**NT**
**TRBP- dsRBDmt1+2**	**complex**	ND	ND	NT	NT	NT	NT	**ND**	NT	NT	**NT**	NT	NT	**NT**
**TRBP- ΔdsRBD3**	**complex 1**	5.00	12.0	NT	NT	NT	NT	**8.50**	NT	NT	**NT**	NT	NT	**NT**
	**complex 2**	180	220	NT	NT	NT	NT	**200**	NT	NT	**NT**	NT	NT	**NT**
**PACT-WT**	**complex 2**	220	401	NT	NT	NT	NT	**311**	222	401	**312**	1030	502	**766**
**PACT- dsRBDmt1**	**complex 2**	3900	1900	NT	NT	NT	NT	**2900**	NT	NT	**NT**	NT	NT	**NT**
**PACT- dsRBDmt2**	**complex 2**	5700	4600	NT	NT	NT	NT	**5150**	NT	NT	**NT**	NT	NT	**NT**
**PACT- dsRBDmt1+2**	**complex**	ND	ND	NT	NT	NT	NT	**ND**	NT	NT	**NT**	NT	NT	**NT**
**PACT- ΔdsRBD3**	**complex 1**	1500	810	NT	NT	NT	NT	**1160**	NT	NT	**NT**	NT	NT	**NT**
	**complex 2**	ND	ND	NT	NT	NT	NT	**ND**	NT	NT	**NT**	NT	NT	**NT**

* and ** indicate 21nt dsRNA with blunt ends and siRNA with 2nt DNAs at both 3′ overhangs, respectively.

ND  =  not determined.

NT  =  not tested.

A supershift analysis was performed to confirm that the complex 1 was indeed formed with ^32^P-labeled siLuc-36 or siLuc2-153, as well as the TRBP-WT protein. The complex 1 (formed at 1.3 nM TRBP-WT protein concentration) was supershifted by anti-myc antibody, which recognizes the C-terminal myc-tag of TRBP-WT protein, but not by control anti-Flag antibody (Figures 1E and S1B), indicating that the complex 1 is composed of ^32^P-labeled siRNA and the TRBP-WT protein. The result that the complex 2 was formed from about 20 nM of TRBP-WT protein suggests that the complex 2 consists of the components contained in the complex 1 and additionally supplied TRBP-WT protein, because TRBP-WT protein was abundant but the majority of free siRNAs were absent when the complex 2 appeared (Figures 1B, S1A and C-F).

**Figure 2:**
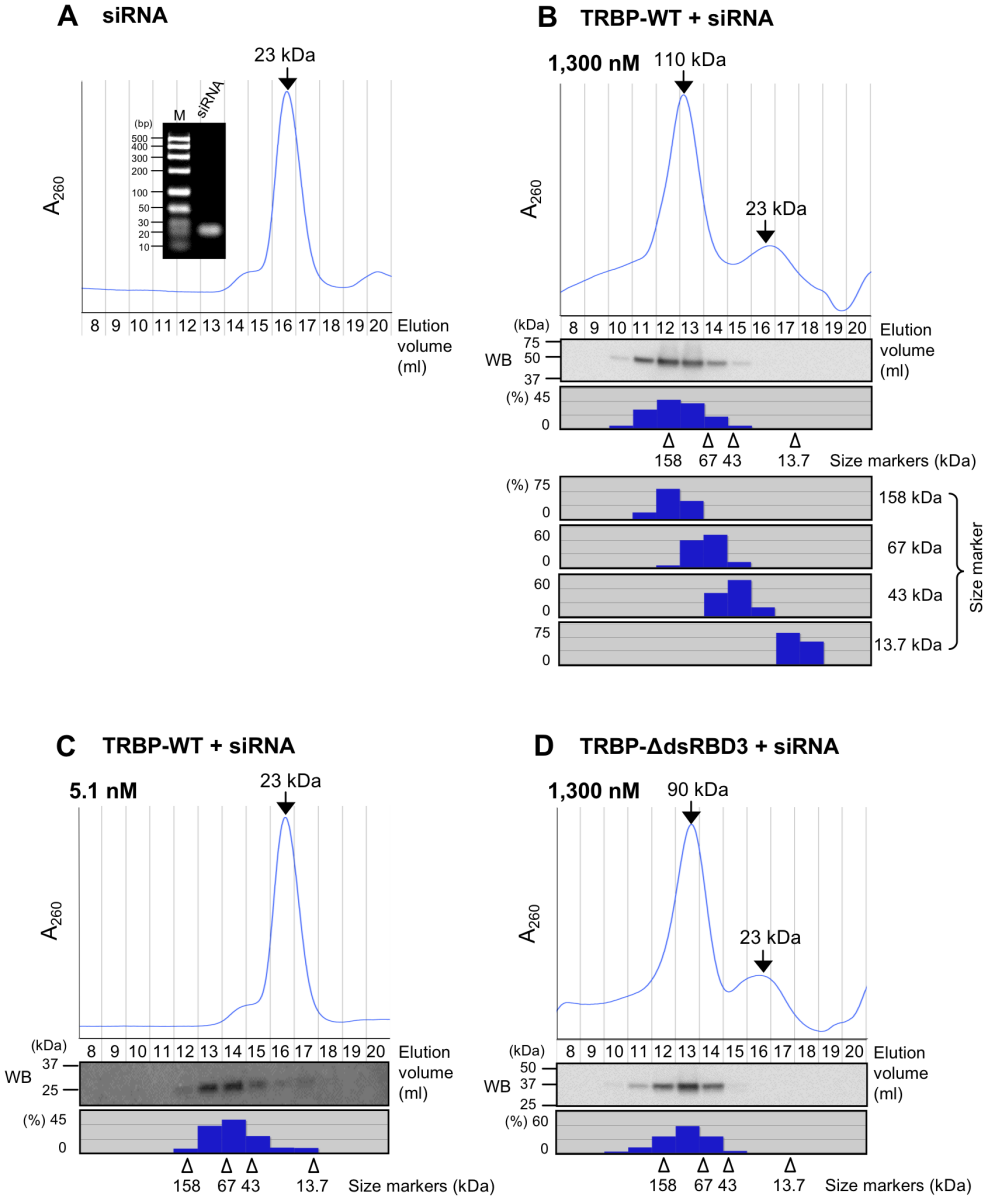
Gel filtration chromatography of purified TRBP-WT and TRBP-ΔdsRBD3 proteins with siRNA. Gel filtration chromatography patterns of non-labeled 300 nM of siLuc-36 alone (A), siLuc-36 with 1,300 nM of TRBP-WT (B), siLuc-36 with 5.1 nM of TRBP-WT(C) and siLuc-36 with 1,300 nM of TRBP-ΔdsRBD3 (D). In A, inset, siLuc-36 was electrophoresed on a 3% agarose gel and stained with EtBr. Lower panels in B-D showed the results of Western blot (WB) by anti-myc antibody for detecting TRBP proteins in the elution fractions. Histograms below WBs showed the quantified signal densities of TRBP proteins detected by WB. Arrowheads indicate the positions of molecular weight size markers. In B, each fraction of molecular weight size markers was quantified and represented in histogram after staining the gel with Coomassie brilliant blue.

To identify components of the complexes 1 and 2 observed in TRBP-WT EMSA experiments, we performed gel filtration analysis using a Superdex 200 HR 10/300 column. Initially, we evaluated the elution pattern of siRNA on the gel filtration column. When free siLuc-36 alone was applied to the column, a single clear peak was detected (Figure 2A). The elution position calculated based on the protein molecular weight markers corresponded to a position of about 23 kDa protein. Because siLuc-36 forms a single siRNA pair (∼14 kDa) by agarose gel electrophoresis (Figure 2A, inset), a 21 nt RNA duplex was likely detected at a molecular weight of about 23 kDa (Figure 2A). When purified TRBP-WT (1,300 nM) was pre-incubated with siRNA (300 nM), two peaks at 110 kDa and 23 kDa were observed (Figure 2B). Because the molecular weight of TRBP-WT containing His- and myc-tags is approximately 44 kDa, the fastest 110 kDa peak of the ribonucleoprotein complex, corresponding to complex 2, may be composed of two molecules of TRBP-WT protein (88 kDa) and a siRNA (23 kDa) pair as predicted by EMSA experiments. This peak was detected between the position of molecular weight size markers of 158 and 67 kDa, but TRBP-WT protein band was observed alone at about 44 kDa by Western blot (Figure 2B), suggesting that TRBP-WT proteins are actually dimerized in the complex. Furthermore, to investigate the component of complex 2 in more detail, non-labeled siRNA at concentrations from 0 to 600 nM was incubated with the complex 2 (Figure 1F). According to the increase of additional siRNA concentrations, the second step band of complex 2 was gradually disappeared and the first step band of complex 1 became prominent. These results suggest that, one molecule of siRNA-free TRBP-WT protein, which may not directly interact with siRNA but dimerized with a TRBP-WT protein, in the complex 2 is capable of binding a siRNA. However, upon the siRNA-free TRBP-WT protein binds to a siRNA, the TRBP-WT homodimers were dissociated into the monomers consisting of the one molecule of TRBP-WT and a siRNA, and free ^32^P-labeled siRNAs were detected from about 600 nM concentration of non-labeled siRNAs at which free siRNAs might be saturated. Thus, the affinity of TRBP with siRNA is stronger than that for own dimerization. The second peak in Figure 2B was found in the same elution position as free siLuc-36, indicating that this peak contains free siRNA alone.

Although the complex 1 is substantially observed by EMSA at TRBP protein concentrations lower than 330 nM (Figure 1B), we could not detect the peak at about 67 kDa, which corresponding to the complex 1, by gel filtration under the concentration of 330 nM TRBP-WT, probably due to the protein concentration is below the detection limit. However, when purified TRBP-WT at a low concentration of 5.1 nM was pre-incubated with siLuc-36, the TRBP-WT protein was detected by Western blot in the fraction 14 which includes the proteins with molecular weight of about 48–75 kDa (Figure 2C). Thus, this complex might be actually complex 1 containing a TRBP monomer bound to the siRNA.

### siRNA binding by TRBP with mutations in dsRBD1 and/or dsRBD2, and deletion of dsRBD3

**Figure 3:**
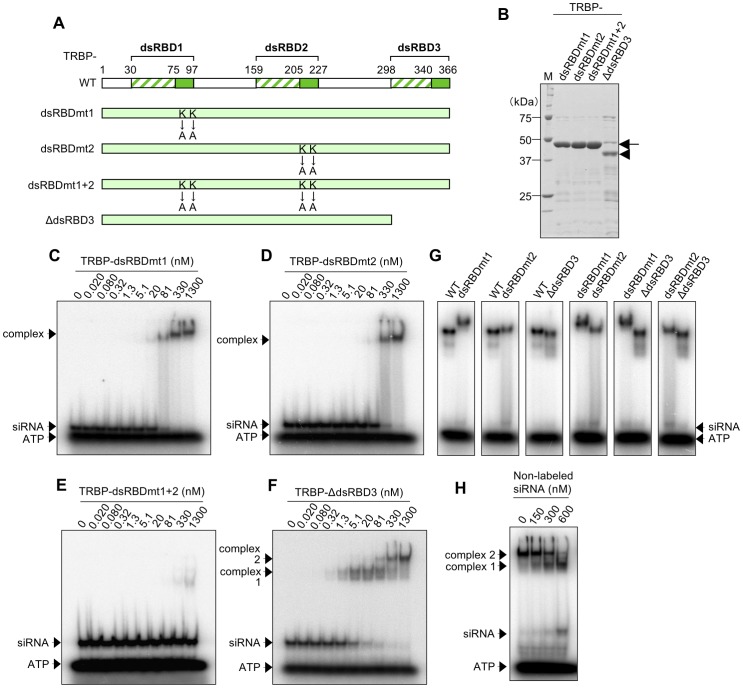
Structure, purification and EMSA of TRBP mutant proteins. (A) Schematic representation of TRBP-WT and its mutant proteins. Amino acids at positions 80 and 81 were substituted from lysines (KK) to alanines (AA) in TRBP-dsRBDmt1, and those at 210 and 211 were also substituted from KK to AA in TRBP-dsRBDmt2. TBRP-dsRBDmt1+2 contains both substitutions. The dsRBD3 was deleted in TRBP-ΔdsRBD3. All mutants were tagged with N-terminal His and C-terminal myc, in the same way as TRBP-WT. (B) Coomassie brilliant blue stained patterns of purified TRBP mutant proteins resolved by SDS-PAGE. Arrows indicate the migration position of TRBP-dsRBDmt1, TRBP-dsRBDmt2, and TRBP-dsRBDmt1+2. Arrowhead indicates TRBP-ΔdsRBD3 protein. M represents the protein size markers. (C–F) EMSA patterns of TRBP-dsRBDmt1 (C), TRBP-dsRBDmt2 (D), TRBP-dsRBDmt1+2 (E) and TRBP-ΔdsRBD3 (F) with ^32^P-labeled siLuc-36. ^32^P-labeled siRNA (0.50 nM) was incubated with increasing amounts of TRBP protein, as indicated. (G) The comparison of mobilities of siRNA complexes with TRBP-WT, TRBP-dsRBDmt1, TRBP-dsRBDmt2, TRBP-dsRBDmt1+2 and TRBP-ΔdsRBD3 proteins (1,300 nM), respectively. (H) EMSA pattern of TRBP-ΔdsRBD3 protein (1,300 nM) mixed with ^32^P-labeled siLuc-36 (0.50 nM) incubated with increasing amount of non-labeled siLuc-36.

The roles of the three TRBP dsRBDs in siRNA binding were examined using purified N-terminal His- and C-terminal myc-tagged TRBP proteins with mutations in dsRBD1 and/or dsRBD2, and a deletion of dsRBD3, designated as TRBP-dsRBDmt1, TRBP-dsRBDmt2, TRBP-dsRBDmt1+2, and TRBP-ΔdsRBD3, respectively (Figure 3A). The homogeneities of these recombinant proteins were examined using SDS-PAGE (Figure 3B). TRBP-dsRBDmt1, in which lysine residues at 80 and 81 in dsRBD1 were replaced with alanines, could not bind to siLuc-36 (Figure 3C) and siLuc2-153 (Figure S2A) at low protein concentration and bound to them in one step at high protein concentration. TRBP-dsRBDmt2 substituted with alanines at positions 210 and 211 also showed one-step binding patterns at high protein concentration (Figures 3D and S2B). TRBP-dsRBDmt1+2 with mutations in both dsRBD1 and dsRBD2 virtually could not bind to siLuc-36 (Figure 3E) or siLuc2-153 (Figure S2C), indicating that dsRBD3 does not play a role in dsRNA binding, as reported previously [25]. The slight binding activities observed at 330 and 1,300 nM of TRBP-dsRBDmt1+2 protein concentrations in Figure 3E might be the results of weak binding activities of mutated dsRBD1/dsRBD2, or dsRBD3. Although we tried to construct the expression construct with mutations in dsRBD1 and dsRBD2 in addition to the deletion of dsRBD3 (TRBP-dsRBDmt1+2Δ3) to investigate the contribution of each dsRBDs to the weak binding of TRBP-dsRBDmt1+2 protein, we could not purify TRBP-dsRBDmt1+2Δ3 protein because of its high instability. TRBP-ΔdsRBD3 showed binding patterns similar to TRBP-WT in two steps from 0.32 and 81 nM, respectively (Figures 3F and S2D). Gel filtration chromatography revealed a primary peak at 1,300 nM TRBP-ΔdsRBD3 protein concentration was detected at 90 kDa (TRBP-ΔdsRBD3 complex 2, Figure 2D). The fastest peak was observed between the position of molecular weight size markers of 158 and 67 kDa, but protein band was observed alone at about 37 kDa by Western blot, indicating that this peak might contain two molecules of TRBP-ΔdsRBD3 (∼37 kDa) and a single siRNA pair, and the ∼27 kDa peak was free siRNA (Figures 2D and 3F). As shown in TRBP-WT (Figure 1F), non-labeled siRNAs at concentrations from 0 to 600 nM was also incubated with TRBP-ΔdsRBD3 complex 1 (Figure 3H). The migrating band of complex 2 was gradually disappeared and that of complex 1 became prominent according to the increase of siRNA concentrations in an analogous fashion with TRBP-WT. Furthermore, free ^32^P-labeled siRNAs were appeared from about 600 nM concentration of non-labeled siRNAs. Thus, one molecule of siRNA-free TRBP-ΔdsRBD3 protein in the complex 2 (Figure 3F) is also capable of binding to a siRNA. However, upon the siRNA-free TRBP-ΔdsRBD3 protein binds to a siRNA, the TRBP-ΔdsRBD3 homodimers were dissociated into the monomers consisting the one molecule of TRBP-ΔdsRBD3 and a siRNA. Their *K*_d_ values calculated from siRNA-binding curves were about 8.50 and 200 nM, respectively (Table 1), similar to the wild type. However, the fastest migrating bands of complex 1 were rather broad, probably because the TRBP-ΔdsRBD3 protein was slightly unstable (see Figure 3B).

Mobilities of the ribonucleoprotein complexes composed of siRNA and TRBP-WT or its dsRBD mutant proteins were compared on a native gel at 1,300 nM concentrations (Figure 3G). Although the molecular weight of TRBP-WT is similar to TRBP-dsRBDmt1 and TRBP-dsRBDmt2, neither complex showed the same mobility, which were rather slow compared to TRBP-WT. This suggests that the complexes containing TRBP-dsRBDmt1 or TRBP-dsRBDmt2 might be multimerized. The EMSA experiments also suggested that TRBP-dsRBDmt1 and TRBP-dsRBDmt2 complexes formed aggregates of large molecular weight (Figures 3C and D, and S2A and B). Gel filtration chromatography revealed that TRBP-dsRBDmt1 and TRBP-dsRBDmt2 readily form aggregates at increasing protein concentrations (Figure S3). Next, the TRBP-dsRBDmt1 and TRBP-dsRBDmt2 proteins at concentrations from 330 to 1,300 nM were incubated with siRNA. The molecular weights of gel filtration peaks increased from 180 to 250 kDa for TRBP-dsRBDmt1 (Figure S3A), and from 120 to 170 kDa for TRBP-dsRBDmt2 (Figure S3B). However, the gel filtration peaks of TRBP-WT and TRBP-ΔdsRBD3 proteins with siRNA did not increase, even when their protein concentration was increased (Figure S3C and D). These results suggest that TRBP-dsRBDmt1 and TRBP-dsRBDmt2 form aggregates more easily than TRBP-WT and TRBP-ΔdsRBD3, possibly because the mutated dsRBD1 and dsRBD2 domains are easy to associate when their interaction with siRNA is abolished.

### Dimerized PACT binds to siRNA at high concentrations

Both PACT and TRBP bind to Dicer, and are thought to be involved in the RNA silencing pathway. Furthermore, the amino acid sequences of dsRBD1, dsRBD2, and dsRBD3 in TRBP share 56%, 59%, and 58% identities and 76%, 83%, and 75% similarities, respectively, with those of the corresponding PACT domains.

**Figure 4:**
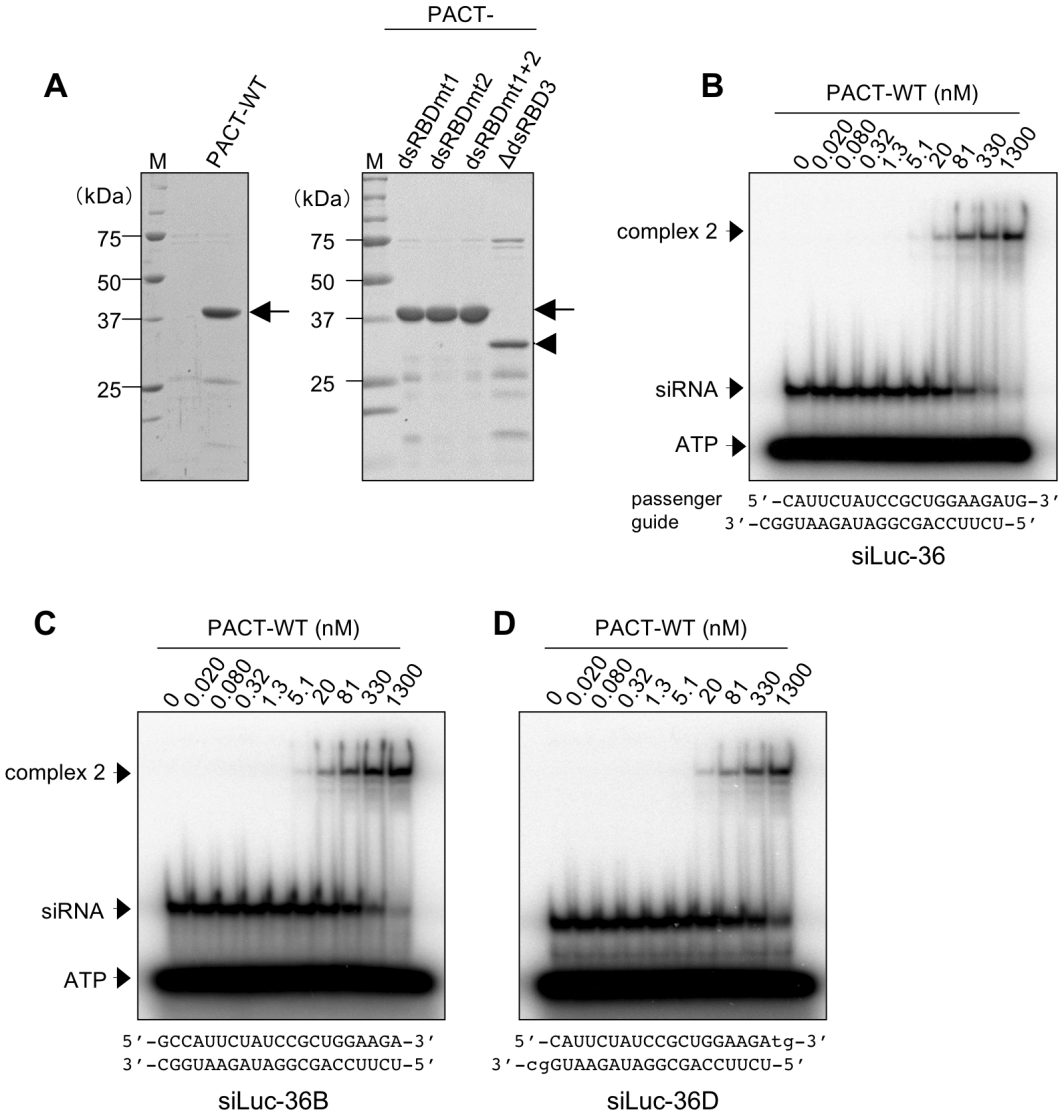
Purification of PACT-WT and its mutant proteins, and EMSA of PACT-WT protein. (A) Coomassie brilliant blue stained pattern of purified PACT-WT (left panel), and its mutant proteins (right panel) resolved by SDS-PAGE. Arrows indicate the migration position of PACT-WT, PACT-dsRBDmt1, PACT-dsRBDmt2, and PACT-dsRBDmt1+2 proteins. Arrowhead indicates PACT-ΔdsRBD3 protein. M represents the protein size markers. (B–D) EMSA patterns of PACT-WT with ^32^P-labeled siLuc-36 (B), siLuc-36B (C), and siLuc-36D (D). ^32^P-labeled siRNA (0.50 nM) was incubated with increasing amounts of PACT-WT as indicated. Lower cases in D indicate DNAs.

To compare the dsRNA binding mode of PACT protein with that of TRBP protein, a recombinant N-terminal His- and C-terminal myc-tagged PACT protein (PACT-WT) was also expressed in *E. coli* and purified by Ni-NTA agarose. Recombinant PACT-WT protein was soluble and the homogeneity was ascertained by SDS-PAGE (Figure 4A). The binding affinity of PACT-WT protein to siRNA was examined by EMSA experiments with ^32^P-labeled siRNA. The single migrating band (referred to as complex 2) was observed starting at 20 nM concentration of PACT-WT protein with siLuc-36 (Figure 4B) or siLuc2-153 (Figure S4A). The siRNA-binding curve derived from the EMSA experiments performed with PACT-WT at concentrations of 0–1,300 nM indicated that the average *K*_d_ value was 331 nM, similar to the second binding step band of TRBP-WT protein (Table 1). As shown in the experiment using TRBP protein (Figure 1C and D), we performed EMSA with 21 nt dsRNA with blunt ends (siLuc-36B and siLuc2-153B) and siRNA containing 2 nt DNAs at both 3′ overhangs (siLuc-36D and siLuc2-153D) to examine the effects of 2 nt single-stranded RNA at 3′ overhangs on the binding activity with PACT protein. Single migrating bands were observed by siLuc-36B and siLuc2-153B and their *K*_d_ values were 222 and 401 nM, respectively, for the formation of complex 2 (Table 1), closely similar to the results of siLuc-36 and siLuc2-153 (Figures 4C and S4B). siLuc-36D and siLuc2-153D also showed similar binding patterns to those of siLuc-36 and siLuc2-153 (Figures 4D and S4C) and their *K*_d_ values were almost similar or slightly high at 502 nM (siLuc2-153D) and 1,030 nM (siLuc-36D), respectively, for the formation of complex 2 (Table 1), suggesting that 2 nt DNAs at 3′ overhangs of siRNAs may not affect the binding activity with PACT, if any.

**Figure 5:**
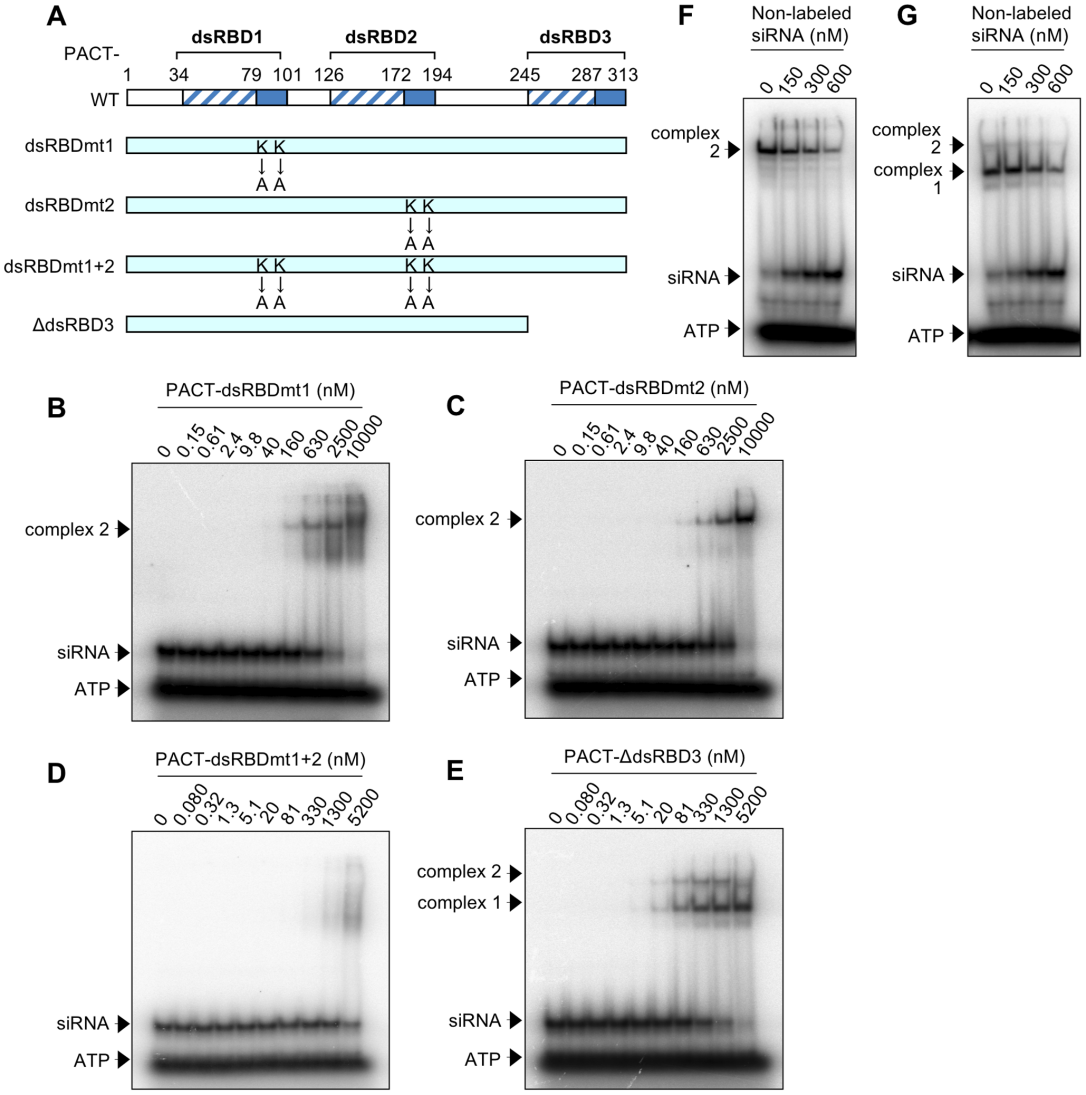
Structure and EMSA of PACT-WT and PACT mutant proteins. (A) Schematic representation of PACT-WT and its mutant proteins. Amino acids at positions 84 and 85 were substituted from lysines (KK) to alanines (AA) in PACT-dsRBDmt1, and those at 177 and 178 were also substituted from KK to AA in PACT-dsRBDmt2. PACT-dsRBDmt1+2 contained both substitutions. The dsRBD3 was deleted in PACT-ΔdsRBD3. All mutants were tagged with N-terminal His and C-terminal myc, in the same way as PACT-WT. (B–E) EMSA patterns of PACT-dsRBDmt1 (B), PACT-dsRBDmt2 (C), PACT-dsRBDmt1+2 (D), and PACT-ΔdsRBD3 (E) with ^32^P-labeled siLuc-36. (F, G) EMSA patterns of PACT-WT (F) and PACT-ΔdsRBD3 (G) (1,300 nM) mixed with ^32^P-labeled siLuc-36 (0.50 nM) incubated with increasing amount of non-labeled siLuc-36.

The role of three dsRBDs in PACT for siRNA binding were examined using purified N-terminal His- and C-terminal myc-tagged PACT protein with mutations in dsRBD1 and/or dsRBD2, or a deletion of dsRBD3, which were termed as PACT-dsRBDmt1, PACT-dsRBDmt2, PACT-dsRBDmt1+2, and PACT-ΔdsRBD3, respectively, as shown in Figure 5A. PACT-dsRBDmt1, in which lysine residues at 84 and 85 in dsRBD1 were replaced with alanines, bound to siLuc-36 and siLuc2-153 in one step from protein concentrations of 160 nM (complexes 2 in Figures 5B and S4D). PACT-dsRBDmt2 replaced with alanines at 177 and 178 also showed a single band from protein concentration of 630 nM (complexes 2 in Figures 5C and S4E) with rather weak affinity compared to PACT-dsRBDmt1. PACT-dsRBDmt1+2 with mutations in both dsRBD1 and dsRBD2 had very low siRNA binding affinity if at all (Figures 5D and S4F), which suggests that dsRBD3 is not responsible for siRNA binding in PACT protein. However, the EMSA experiment using PACT-ΔdsRBD3 protein represented that two different migrating bands appeared simultaneously at 81 nM, and the fastest band (complex 1) was predominant, and the slowest band (complex 2) was subsidiary (Fig. 5E and S4G). Their average *K*d values were 1,160 nM (Table 1). The mobility of the ribonucleoprotein complexes composed of siRNA and PACT-WT or its dsRBD mutant proteins were compared on a native gel at protein concentrations of 1,300 nM (Figure S4H). The mobilities of the complexes 2 composed of PACT-WT, PACT-dsRBDmt1, and PACT-dsRBDmt2 with siRNA were similar. However, the mobility of complex 1 containing PACT-ΔdsRBD3 protein with siRNA was different to that of complex 2 of PACT-WT and the other mutant proteins, and the mobility of PACT-ΔdsRBD3 complex 2 was similar to them.

**Figure 6:**
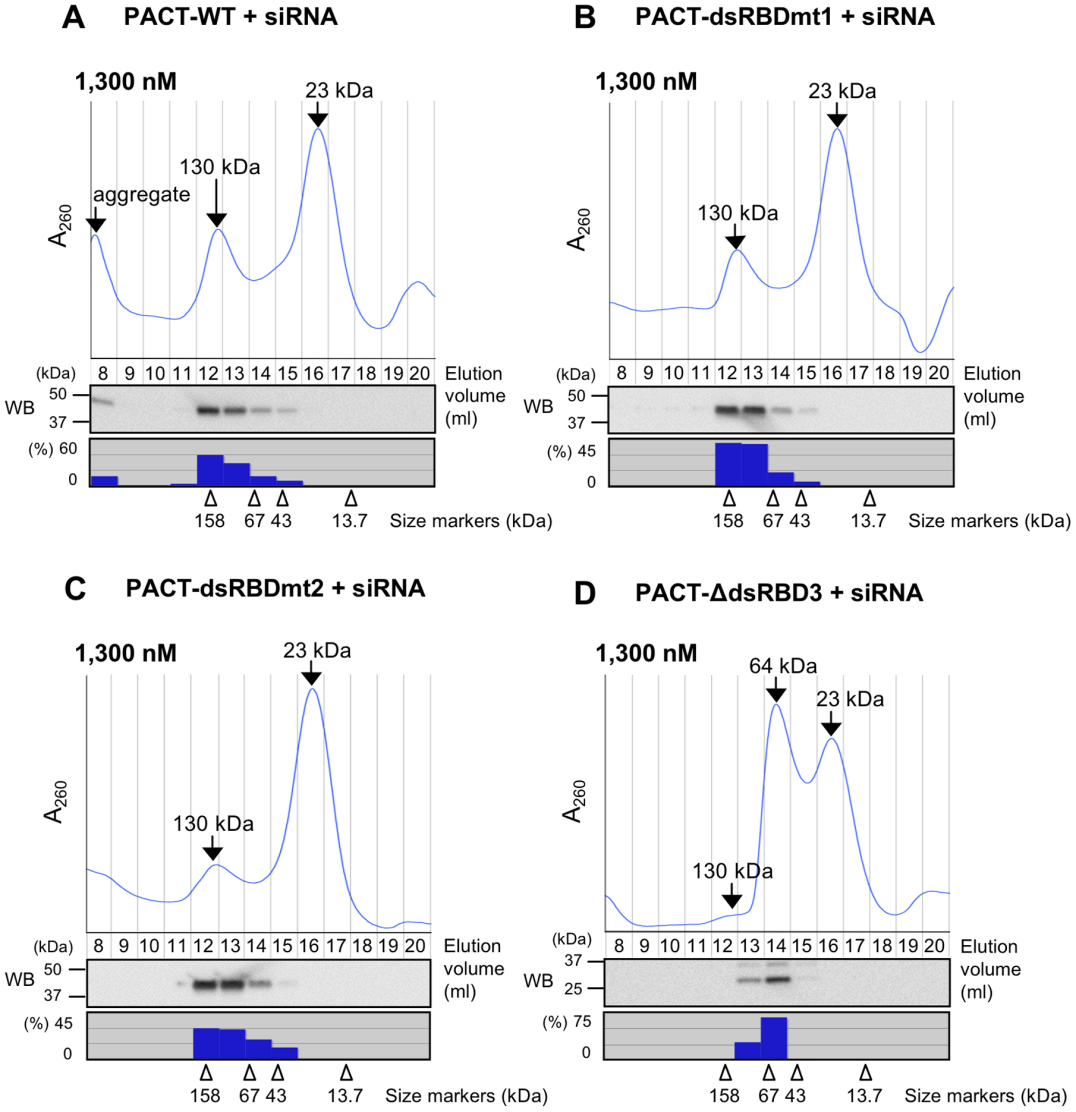
Gel filtration chromatography of purified PACT-WT and PACT mutant proteins with siRNA. Gel filtration chromatography patterns of non-labeled siLuc-36 (300 nM) with PACT-WT (A), PACT- dsRBDmt1 (B), PACT-dsRBDmt2 (C), and PACT-ΔdsRBD3 (D). Lower panels showed the results of Western blot (WB) by anti-myc antibody for detecting PACT proteins in the elution fractions. Histograms below WBs showed the quantified signal densities of PACT proteins detected by WBs. Arrowheads indicate the positions of molecular weight size markers.

Gel filtration chromatography revealed that PACT-WT, PACT-dsRBDmt1 and TRBP-dsRBDmt2 proteins formed complexes with similar molecular weights of 130 kDa (Figure 6A–C). Although these fastest peaks, probably corresponding to complexes 2, were observed between the positions of molecular weight size markers of 158 and 67 kDa, but protein bands detected by SDS-PAGE were shown at about 40 kDa (Figures 4A and 6A–D), indicating that more than one molecules of PACT-WT proteins are contained in the complex. The molecular weight of PACT-WT, PACT-dsRBDmt1 and PACT-dsRBDmt2 proteins are approximately 40 kDa, so it is possible that two molecules of each of PACT-WT, PACT-dsRBDmt1 and PACT-dsRBDmt2 proteins (∼80 kDa) and two molecules of siRNAs (46 kDa) are contained in the complex 2. However, calculation of the number of PACT-WT or PACT-ΔdsRBD3 proteins interacted with one molecule of siRNA using their molar absorptivities suggested that the two molecules of PACT-WT proteins interact with one molecule of siRNA in complex 2. Thus, we could not determine the accurate number of siRNA molecules contained in the complex 2. The PACT-WT dimer in the complex 2 may interact with one or two molecules of siRNA(s). The PACT-ΔdsRBD3 mutant protein and siRNA showed two major peaks, and the molecular weight of the fastest major peak was 64 kDa, indicating that the complex might be composed of one molecule of 33 kDa of PACT-ΔdsRBD3 and a 23 kDa siRNA, while the second peak represented free siRNA (Figure 6D). In addition, a negligible small peak was also observed at about 130 kDa. This band might probably correspond to the complex 2 in Figure 5E. Thus, the complex 1 is ascertained to be a major complex of the PACT-ΔdsRBD3 mutant protein and siRNA.

As shown in TRBP-WT and TRBP-ΔdsRBD3 proteins, PACT-WT and PACT-ΔdsRBD3 proteins at 1,300 nM were incubated with non-labeled siRNA at concentrations from 0 to 600 nM after incubation with ^32^P-labeled siRNA (0.50 nM) (Figure 5F and G). Unlike TRBP, the amounts of complex 2 in Figure 5F, and complexes 1 and 2 in Figure 5G gradually decreased, whereas the amount of free siRNA increased, according to the increase of non-labeled siRNA concentrations. The results suggest that the complex 2 of PACT-WT or PACT-ΔdsRBD3 is already filled with siRNA: the PACT-WT complex 2 includes two molecules of PACT proteins and each of them might contain a siRNA, while PACT-ΔdsRBD3 complex 1 composed of one molecule of PACT-ΔdsRBD3 and a siRNA. Then, only the replacements of ^32^P-labeled siRNA to non-labeled siRNA occurred in the complexes 1 and 2 according to the increase of additional non-labeled siRNA concentrations. Alternatively, the complex 2 might contain two molecules of PACT-WT proteins and one molecule of siRNA. However, because PACT-WT dimer constructed very stable dimer formation and cannot incorporate siRNA without dissociating into monomer, no additional siRNA could not be incorporated into the complex 2. The dsRBD3 domain, which is one of the essential regions for dimerization, was deleted in the PACT-ΔdsRBD3 protein, so their dimerized form might be very unstable, and equilibrium state of complexes 1 and 2 observed in Figure 5E showed that complex 1 is more stable compared to complex 2. Thus, these results suggest that the intermolecular interactions due to dsRBD3 in PACT proteins are stronger compared to the interaction between PACT and siRNA.

## Discussion

Our results clearly show that TRBP-WT and PACT-WT proteins bind siRNA, although their binding patterns based on EMSA differed (Figures 1B, 4B, S1A, C-F and S4A). The dsRBD1 or dsRBD2 mutant, and the dsRBD deletion mutant (TRBP-dsRBDmt1, TRBP-dsRBDmt2, and TRBP-ΔdsRBD3, PACT-dsRBDmt1, PACT-dsRBDmt2, and PACT-ΔdsRBD3) also bound siRNA (Figures 3C, D, F, 5B, C, E, S2A, B, D, S4D, E and G). However, TRBP-dsRBDmt1+2 with mutations in both dsRBD1 and dsRBD2 showed little binding activities to siRNA if at all (Figures 3E and S2C), as shown in the previous report [17], [19]. We revealed that PACT-dsRBDmt1+2 also showed little binding activities to siRNA (Figures 5D and S4F), indicating that PACT proteins also bind siRNA with dsRBD1 and dsRBD2, while dsRBD3 is not involved in siRNA binding. The mean *K*_d_ values of TRBP-dsRBDmt1 and TRBP-dsRBDmt2 binding to siRNA were 255 and 445 nM, respectively, and those of PACT-dsRBDmt1 and PACT-dsRBDmt2 were 2,900 and 5,150 nM, respectively (Table 1), indicating that dsRBD2 has a higher affinity to siRNA than dsRBD1.

TRBP-WT bound siRNA in two sequential steps (Figures 1B and S1A). During the first binding step, TRBP-WT formed complex 1 with siRNA at high affinities at concentrations as low as 0.32 nM. The TRBP-WT complex 2 formed at lower affinities at 20 nM protein concentrations. According to the increase in TRBP-WT concentration, the amount of complex 1 gradually decreased while complex 2 increased. Gel filtration chromatography and Western blot analysis suggested that complex 1 consisted of monomeric TRBP-WT, which binds a siRNA (Figures 1B, 2C and S1A), while complex 2 composed of two molecules of TRBP-WT, either one of TRBP-WT proteins is considered to bind a single molecule of siRNA, although we cannot omit the possibility that TRBP binds to two separate sites on the siRNA. Interestingly, when increasing amounts of siRNAs were added to the complex 2, the migrating band of TRBP-WT protein was shifted to the position of complex 1, suggested that the siRNA-free TRBP-WT protein contained in the homodimer of complex 2 dissociated to the monomers of complex 1 by incorporating siRNA (Figure 1F). TRBP is known to form homodimers via dsRBD1 and dsRBD2 without dsRBD3 [14]. Our results suggested that the affinity of TRBP binding to siRNA is higher than its affinity for homodimerization. Their *K*d values were 5.30∼33.0 nM for complex 1 and 44.0∼440 nM for complex 2 (Table 1). Although these values were slightly higher than previously reported values of 0.24 [19] or 0.77 [26] nM for complex 1 equivalent and 13.3 nM [19] for complex 2 equivalent, this may be derived from the different experimental conditions such as protein preparation, buffer composition, experimental method (EMSA or isothermal titration calorimetry), protein tags (His/Myc-tag, His-tag, or MBP-tag), and/or siRNA sequence.

PACT-WT protein bound to siRNA in one step (Figures 4B and S4A). PACT-WT could not form a complex with siRNA at low protein concentrations, but formed complexes at high concentrations starting from 20 nM (PACT-WT complex 2). This complex was likely to be formed with dimeric PACT-WT protein binding to one or two siRNA molecule(s) by gel filtration chromatography and Western blot analysis (Figure 6A), suggesting that affinity of PACT-WT dimerization is higher than that of siRNA binding affinity. However, when dsRBD3 was deleted, the PACT-ΔdsRBD3 protein formed two complexes at high protein concentrations starting from 20 nM (Figures 5E and S4G). Because the molecular weight of the PACT-ΔdsRBD3 complex 1 was about 64 kDa (Figure 6D), this complex represent monomeric PACT-ΔdsRBD3 protein (33 kDa) binding to one molecule of siRNA, while the slowest PACT-ΔdsRBD3 complex 2 represent the dimeric form binding one or two siRNA molecules (Figure 5E). Although dsRBD3 was not essential for siRNA binding, dsRBD3 is important for PACT homodimerization as shown previously [27]. Because the monomeric form (PACT-ΔdsRBD3 complex 1) was predominant (see Figures 5E and S4G), siRNA binding of monomeric PACT-ΔdsRBD3 might be more stable than dimeric PACT-ΔdsRBD3 binding to siRNA.

The fastest migrating complex 1, shown in EMSA of TRBP-WT and siRNA (Figure 1B), was not observed when TRBP-dsRBDmt1 and TRBP-dsRBDmt2 were used (Figure 3C and D). This suggests that both dsRBD1 and dsRBD2 in TRBP bind siRNA simultaneously in the TRBP-WT complex 1, while the mutated dsRBD1 and dsRBD2 of TRBP could not bind siRNA. As shown in Figure S3, TRBP-dsRBDmt1 and TRBP-dsRBDmt2 readily form aggregates at increasing protein concentrations. The molecular weights of aggregated protein sizes were larger for TRBP-dsRBDmt1 compared to TRBP-dsRBDmt2 at the same concentration, indicating that the interaction in dsRBDmt1 domains might be more stable than that in the TRBP-dsRBDmt2 domains.

PACT-dsRBDmt1 and PACT-dsRBDmt2 formed a complex with siRNA in EMSA (PACT-dsRBDmt1 complex 2 and PACT-dsRBDmt2 complex 2) at high protein concentrations starting from 160 nM (Figure 5B and C). However, PACT-WT formed PACT-WT complex 2 at concentrations as low as 5.1 nM (Figure 4B), suggesting that both dsRBD1 and dsRBD2 in PACT protein bind siRNA simultaneously in the PACT-WT complex 1, while the mutated dsRBD1 and dsRBD2 of PACT could not bind siRNA, similar to TRBP. However, PACT-dsRBDmt1 and PACT-dsRBDmt2 did not form large aggregates (Figure 6B and C) even when the protein concentration increased, unlike the TRBP-dsRBDmt1 and TRBP-dsRBDmt2 proteins. This suggests that the PACT homodimer is very stable probably due to the rigid interaction via dsRBD3 domains.

**Figure 7:**
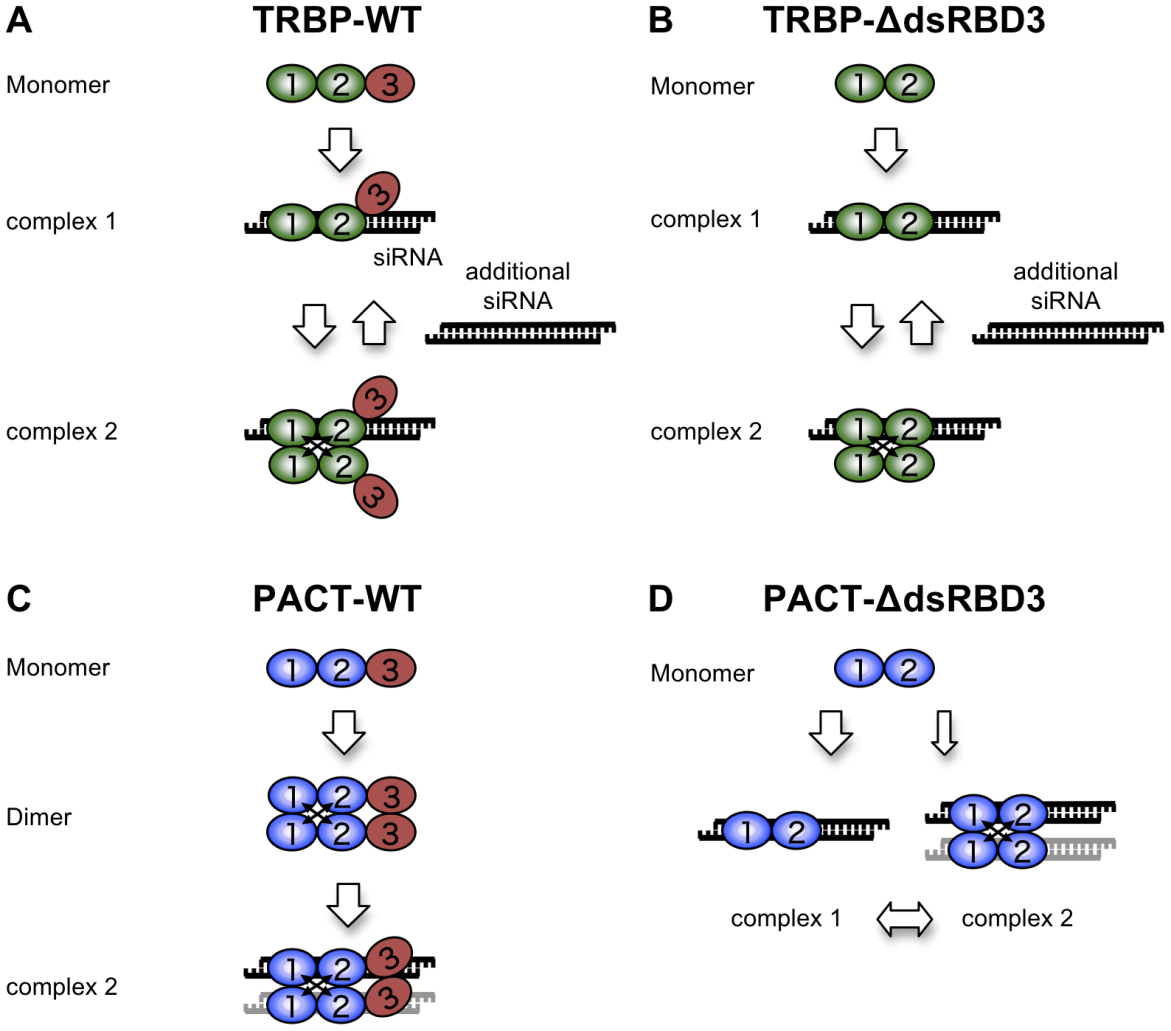
Model of TRBP and PACT binding to siRNA. (A, B) TRBP-WT (A) or TRBP-ΔdsRBD3 (B) binds one molecule of siRNA as a monomer at low concentrations, and then each protein dimerizes due to the increased protein concentration. However, excessive amount of siRNAs were added, TRBP-WT or TRBP-ΔdsRBD3 dimer was dissociated into monomeric TRBP-WT or TRBP-ΔdsRBD3 dimer containing a single molecule of siRNA. (C) PACT-WT forms homodimers at high concentrations and binds to one or two molecules of siRNA. (D) PACT-ΔdsRBD3 binds one siRNA molecule as a monomer or binds one or two siRNA molecules as a dimer. The monomer and dimer may achieve equilibrium, although the monomeric form is predominant. In C and D, we could not determine whether the siRNA shown in gray is contained in the dimerized PACT proteins or not.

We found that the binding modes of human TBRP and PACT to siRNA are unique, although they have similar structural features and contain three dsRBDs (Figure 7). Monomeric TRBP protein can bind siRNA with dsRBD1 and dsRBD2 (Figure 7A). However, at higher concentrations of TRBP-WT proteins, TRBP-WT formed dimers in which one molecule of proteins probably did not interact with siRNA. However, when excess amounts of siRNAs were added, TRBP-WT dimer was dissociated into monomeric form containing a single molecule of siRNA. Thus, TRBP-WT have the higher affinity with siRNA compared to the affinity of own dimerization. A similar binding pattern was also observed in the TRBP protein lacking dsRBD3, namely, TRBP-ΔdsRBD3 (Figure 7B). In contrast, because PACT-WT protein did not bind siRNA as a monomer, PACT is likely to have the higher dimerization affinity compared to that for siRNA-binding via a stable interaction between own dsRBD3s (Figure 7C). However, PACT protein lacking dsRBD3 can bind siRNA as either a monomer or dimer (Figure 7D), similar to TRBP-ΔdsRBD3 protein. In addition, the affinity of siRNA binding of TRBP-ΔdsRBD3 (mean 8.50 nM, Table 1) was considerably higher compared to that of PACT-ΔdsRBD3 (mean 1,160 nM), indicating that the siRNA binding affinities of dsRBD1 and dsRBD2 of TRBP protein are significantly strong. Thus, TRBP may be readily incorporated into the gene-silencing pathway triggered by small RNAs.

*In vivo*, the TRBP-PACT-Dicer interactions (or other factors) may regulate the affinity of siRNA binding of TRBP and PACT under various circumstances. Although TRBP and PACT are known to directly interact with Dicer [14], monomeric TRBP binding and dimeric PACT binding to siRNA may result in the differences in the RISC-loading step of small RNA, the formation and/or stability of RLC, or the cleavage reaction of small RNA by Dicer [28], [29]. Furthermore, phosphorylation of serine 287 in PACT increases its affinity for PKR by weakening PACT's interaction with TRBP. PKR activation by PACT leads to phosphorylation of translation initiation factor eIF-2α, which reduces protein synthesis [30]–[32]. Phosphorylation of TRBP mediated by the mitogen-activated protein kinase (MAPK) Erk is known to enhance miRNA production by increasing the stability of the Dicer-TRBP complex [33]. These factors may regulate the siRNA binding affinity of TRBP and PACT *in vivo*.

In this study, we performed the most simple experiments using siRNAs with completely-matched base-pairings to examine the differences in the binding modes of TRBP and PACT proteins in comparison. Although we did not carry out the experiments for investigating TRBP and PACT binding to miRNAs and hairpin precursor miRNAs, the results might have important biological significance. Our results could offer a suggestion that the binding modes of TRBP and PACT to miRNAs, which often contain bulges or internal loops, might be different from those to siRNA, since TRBP and PACT proteins could not bind to single-stranded RNAs. TRBP is shown to affect the kinetics or sites of precursor miRNA processing by Dicer in RNA-structure-specific manner [29]. It is likely that these effects might be the results of different binding modes of TRBP depending on the structures of miRNAs.

## Materials and Methods

### Oligonucleotides

Guide strand and passenger strand RNA oligonucleotides for EMSA and gel filtration experiments were chemically synthesized (Proligo), as shown in Table S1, and purified by polyacrylamide gel electrophoresis. The guide strand was labeled with [γ^32^P] ATP (6,000 Ci/mmol; Perkin Elmer). Hybridizations were performed by incubating at 95°C for 5 min, and annealed products were examined using 15% polyacrylamide gel electrophoresis in TBE buffer, which can separate 21 bp double-stranded siRNA from 21 nt single-stranded RNA. Almost all RNA was recovered as dsRNA.

### Plasmid construction

Fragment encoding a full length human TRBP was amplified using a cDNA mixture of total RNA extracted from human HeLa cells by PCR with primers containing restriction enzyme sites of HindIII and NotI. The amplified fragment was digested with the same enzyme sites and cloned into pcDNA3.1-myc/His A vector (Invitrogen). C-terminal myc-tagged TRBP fragment was amplified from pcDNA3.1-TRBP using primer with restriction enzyme sites NheI and HindIII. The amplified fragments were digested with same enzyme sites and cloned into pET-28a vector (Novagen).

The PACT CDS was also amplified from a cDNA mixture of total RNA extracted from human HeLa cells by PCR using primers with restriction enzyme sites of BamHI and SalI. The amplified fragment was digested with same enzyme sites and cloned into pET-28a vector amplified using primers with same enzyme sites.

For the mutational analysis of each dsRBDs of TRBP and PACT, the site-directed mutagenesis was carried out. The lysine residues at 80 and 81 in TRBP-dsRBD1 and 210 and 211 in TRBP-dsRBD2 were replaced with alanines in accord with the previous reports [17]–[19]. The lysine residues at 84 and 85 in PACT-dsRBD1 and 177 and 178 in PACT-dsRBD2, corresponding positions mutated in TRBP-dsRBD1 and TRBP-dsRBD2, were replaced with alanines.

The used primers were shown in Table S2.

### Protein purification

The TRBP and PACT expression plasmids were transformed into *E. coli* Rosetta (DE3) pLysS (Novagen), and cultured to an OD_600_ of 0.6. After 6 h of induction in 0.3 mM isopropyl β-d-thiogalactoside, cells were lysed by sonication and the hexa-histidine-fusion proteins were purified with Ni-NTA agarose (QIAGEN) according to the manufacturer's instruction. The samples were exchanged to the buffer containing 20 mM Tris (pH8.0) and 300 mM NaCl using PD-10 Desalting columns (GE healthcare), and determined the concentration using Protein Assay (BIO-RAD).

### Electrophoresis mobility shift assay

EMSAs were performed in binding buffer with 20 mM Tris (pH7.5), 150 mM NaCl, 2 mM EDTA, 1 mM 2-mercaptoethanol, 1 mM dithiothreitol (DTT), 50 ng/µl sonicated salmon sperm DNA, and 0.4 U/µl RNasin (Promega). The purified fusion-proteins were incubated with siRNA containing ^32^P-labeled guide strand (0.5 nM) for 30 min on ice, and the samples were electrophoresed on a 9% polyacrylamide gel and analyzed quantitatively using FLA-2000 image analyzer (Fujifilm). For the supershift analysis, anti-myc antibody or anti-Flag antibody (Cell Signaling) were pre-incubated with purified proteins for 1 h on ice and incubated with ^32^P-labeled siRNAs for 30 min on ice, then analyzed by polyacrylamide gel electrophoresis.

### Gel filtration chromatography

Gel filtration chromatography was performed in gel filtration buffer with 20 mM Tris (pH 7.5), 150 mM NaCl, 2 mM EDTA, 1 mM 2-mercaptoethanol, and 1 mM DTT. The purified fusion-proteins were incubated with chemically synthesized siRNA (300 nM) for 30 min on ice, and samples were applied to a Superdex 200 HR 10/30 column (GE healthcare) equilibrated in gel filtration buffer. The amount of siRNA was quantified by measuring the radioactive intensity of the migrated band separated in PAGE. The protein molecular weight markers used were as follows: 232 kDa, catalase; 158 kDa, aldolase; 67 kDa, albumin; 43 kDa, ovalbumin; 13.7 kDa, ribonulease A.

### Western blot

The elution fractions of gel filtration chromatography was mixed with the equal volume of 2 × SDS PAGE Sample Buffer (4% SDS, 0.1 M Tris-hydrochloric acid (HCl), [pH6.8], 12% 2-mercaptoethanol, 20% glycerol, 0.01% bromophenol blue). After boiling for five minutes, the sample was separated by SDS-PAGE and transferred to PVDF membrane using Trans-Blot Turbo Transfer System (BIO-RAD). The membrane was blocked for 1 hour in TBS-T (20 mM Tris-HCl [pH7.5], 150 mM NaCl, 0.2% Triton X-100) supplemented with 5% DifcoTM Skim Milk (Becton, Dickinson and Company), and incubated with ×2,000-diluted anti-myc antibody (Cell Signaling) at 4°C overnight. The membrane was washed three times with TBS-T, and reacted with ×20,000-diluted HRP-linked anti-rabbit antibody (GE healthcare) at room temperature for 1 h. After being washed three times with TBS-T, the membrane was reacted with ECL Prime Western Blotting Detection Reagent (GE healthcare), and visualized with LAS3000 (FUJIFILM). Each band was quantified with Image Gauge 4.0 (FUJIFILM).

## Supporting Information

Figure S1 **EMSAs of TRBP-WT protein with various siRNAs, dsDNA, and ssRNA.** (A, C–L) The results of EMSAs of TRBP-WT protein, with ^32^P-labeled siRNA siLuc2-153 (A), siLuc-30 (C), siLuc-48 (D), siLuc-18 (E), or siLuc-1430 (F), dsDNAs, dsDNA_Luc-36 (G), dsDNA_Luc2-153 (H), ssRNA_Luc-36 (I), ssRNA_Luc2-153 (J), siLuc2-153B (K), and siLuc2-153D (L). ^32^P-labeled siRNA (0.50 nM) was incubated with increasing amounts of each protein as indicated. (B) Supershift analysis of TRBP-WT (1.3 nM) with no antibodies, control anti-Flag, and anti-myc antibodies. Lowercases in siLuc2-153 sequence indicate DNAs. Arrows in A–F, K, and L indicate positions of complexes 1, complexes 2, and supershifted complex, in addition to siRNA and ATP.
(TIFF)

Figure S2 **EMSAs of TRBP mutant proteins.** EMSA patterns of TRBP-dsRBDmt1 (A), TRBP-dsRBDmt2 (B), TRBP-dsRBDmt1+2 (C), and TRBP-ΔdsRBD3 (D) with ^32^P-labeled siLuc2-153 (0.50 nM), incubated with increasing amounts of TRBP mutants, as indicated. Arrows indicate positions of the first and second step migrating complexes 1 and 2 and supershifted complex, in addition to siRNA and ATP.
(TIFF)

Figure S3 **Gel filtration chromatography of purified TRBP-dsRBDmt1, TRBP-dsRBDmt2, TRBP-WT, and TRBP-**Δ**dsRBD3 proteins with siRNA.** Gel filtration chromatography patterns of non-labeled siLuc-36 (300 nM) with increasing amounts of TRBP-dsRBDmt1 (A) and TRBP-dsRBDmt2 (B) (330, 650, 1,300 nM), TRBP-WT (C) and TRBP-ΔdsRBD3 (D) (650, 1,300 nM). Red lines indicate the positions of siRNA peaks. Blue dotted lines, the peaks of TRBP-dsRBDmt1 (A), TRBP-dsRBDmt2 (B), TRBP-WT (C), and TRBP-ΔdsRBD3 (D) with 330 nM siRNA. The shifted areas of these peaks in A and B were represented as blue dashed lines. Note that the peaks in C and D were not shifted even when these protein concentrations increased.
(TIFF)

Figure S4 **EMSA patterns of PACT-WT and its mutant proteins.** (A–G) EMSA patterns of PACT-WT with ^32^P-labeled siLuc2-153 (A), siLuc2-153B (B), and siLuc2-153D (C), and those of PACT-dsRBDmt1 (D), PACT-dsRBDmt2 (E), PACT-dsRBDmt1+2 (F), and PACT-ΔdsRBD3 (G) with ^32^P-labeled siLuc2-153. ^32^P-labeled siRNA (0.50 nM) was incubated with increasing amounts of PACT-WT and its mutant proteins, as indicated. Lower cases in C in siLuc2-153D sequence indicate DNAs. Arrows indicate positions of the migrating complexes 1 and 2, in addition to siRNA and ATP. (H) Comparison of siRNA complex mobilities of PACT-WT, PACT-dsRBDmt1, PACT-dsRBDmt2, PACT-dsRBDmt1+2 and PACT-ΔdsRBD3 protein (1,300 nM).
(TIFF)

Table S1 **siRNA sequences used in this study.**
(TIFF)

Table S2 **PCR primers used in this study.**
(TIFF)
