# Supplementary figures and images for: Distinguishable In Vitro Binding Mode of Monomeric TRBP and Dimeric PACT with siRNA
Source: PLoS One. 2013 May 2;8(5):e63434. doi: 10.1371/journal.pone.0063434 (PMC3642127; doi:10.1371/journal.pone.0063434)

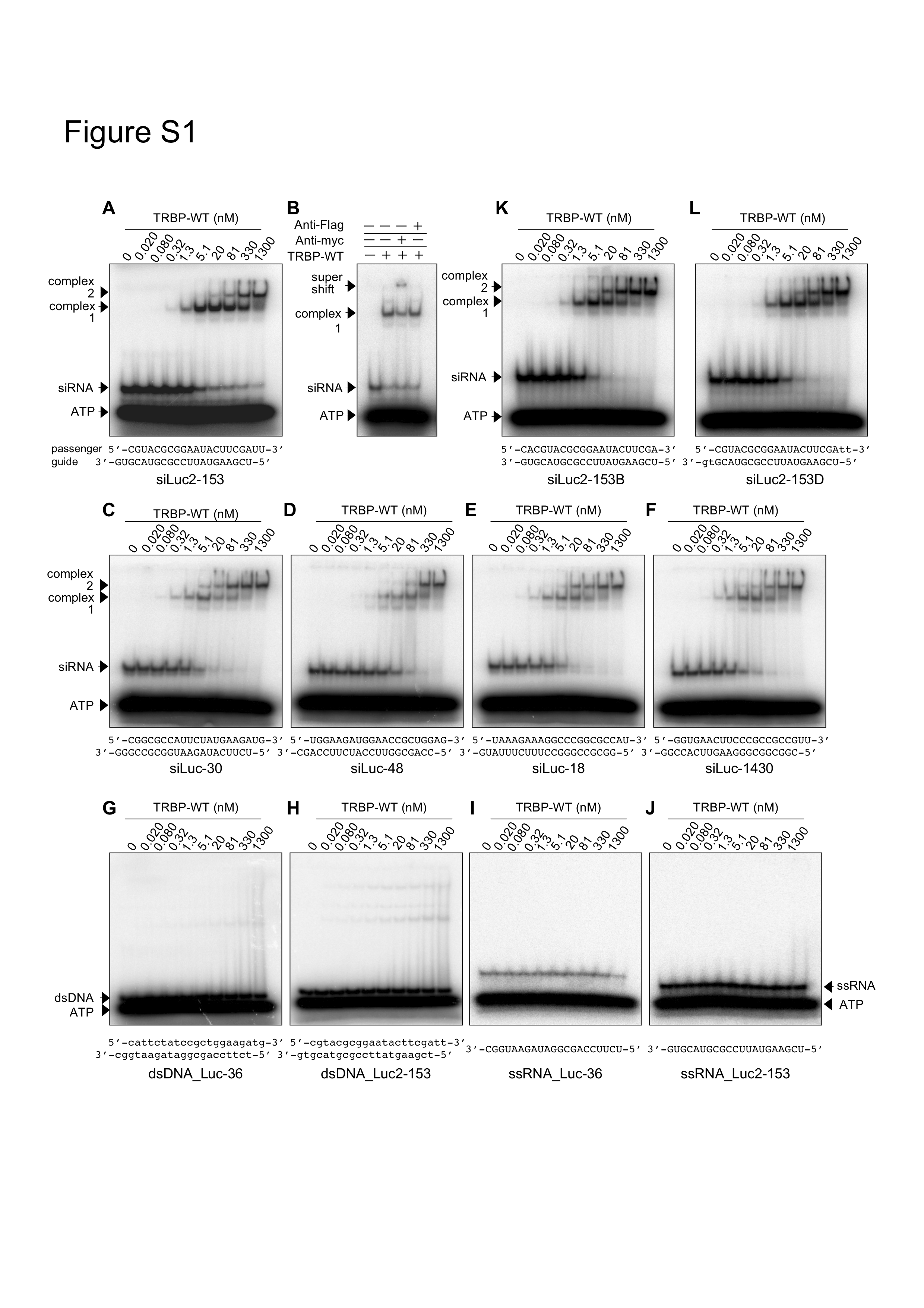

Supplement: Figure S1 — EMSAs of TRBP-WT protein with various siRNAs, dsDNA, and ssRNA. (A, C–L) The results of EMSAs of TRBP-WT protein, with 32P-labeled siRNA siLuc2-153 (A), siLuc-30 (C), siLuc-48 (D), siLuc-18 (E), or siLuc-1430 (F), dsDNAs, dsDNA_Luc-36 (G), dsDNA_Luc2-153 (H), ssRNA_Luc-36 (I), ssRNA_Luc2-153 (J), siLuc2-153B (K), and siLuc2-153D (L). 32P-labeled siRNA (0.50 nM) was incubated with increasing amounts of each protein as indicated. (B) Supershift analysis of TRBP-WT (1.3 nM) with no antibodies, control anti-Flag, and anti-myc antibodies. Lowercases in siLuc2-153 sequence indicate DNAs. Arrows in A–F, K, and L indicate positions of complexes 1, complexes 2, and supershifted complex, in addition to siRNA and ATP. (TIFF) [file pone.0063434.s001.tiff]

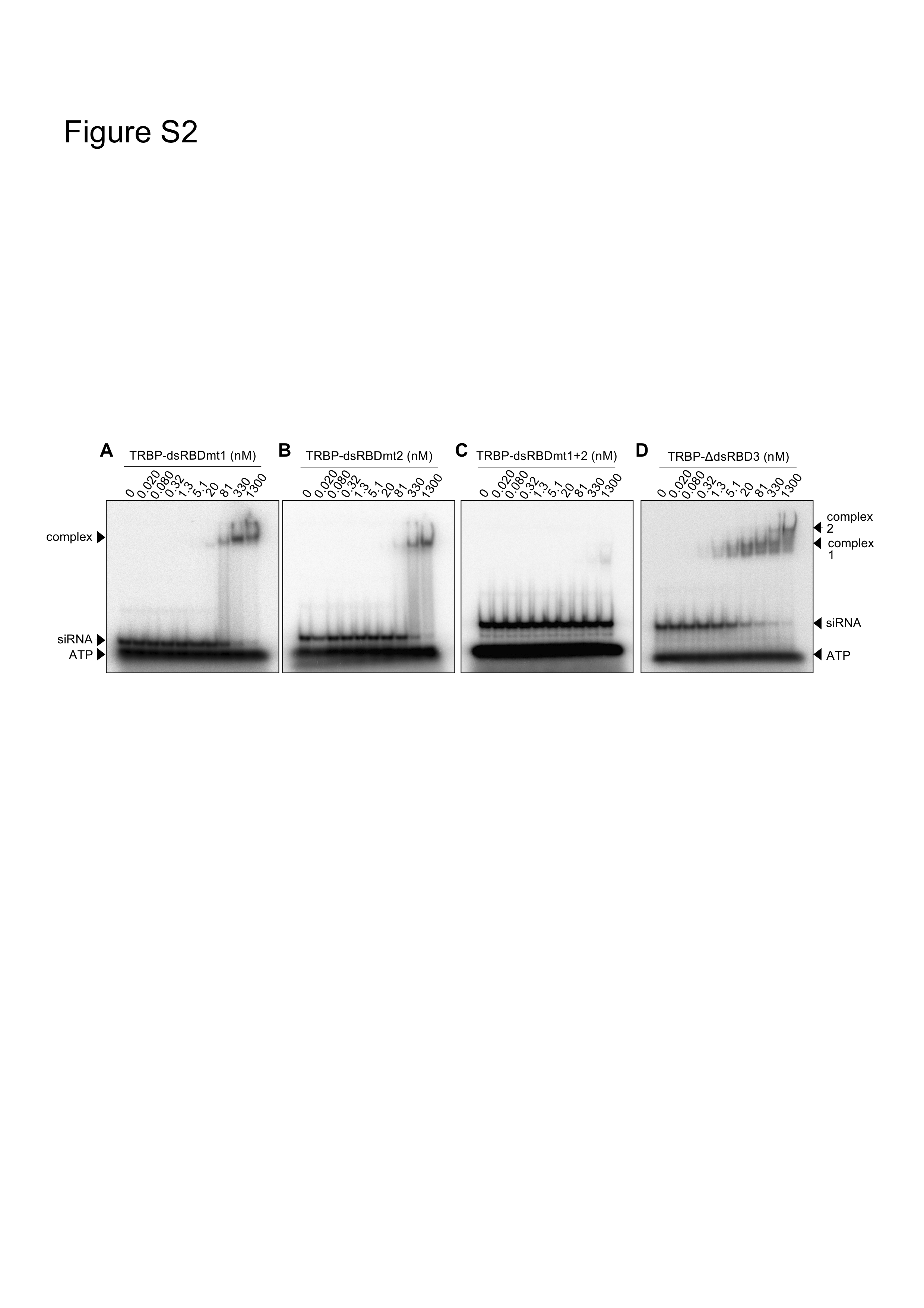

Supplement: Figure S2 — EMSAs of TRBP mutant proteins. EMSA patterns of TRBP-dsRBDmt1 (A), TRBP-dsRBDmt2 (B), TRBP-dsRBDmt1+2 (C), and TRBP-ΔdsRBD3 (D) with 32P-labeled siLuc2-153 (0.50 nM), incubated with increasing amounts of TRBP mutants, as indicated. Arrows indicate positions of the first and second step migrating complexes 1 and 2 and supershifted complex, in addition to siRNA and ATP. (TIFF) [file pone.0063434.s002.tiff]

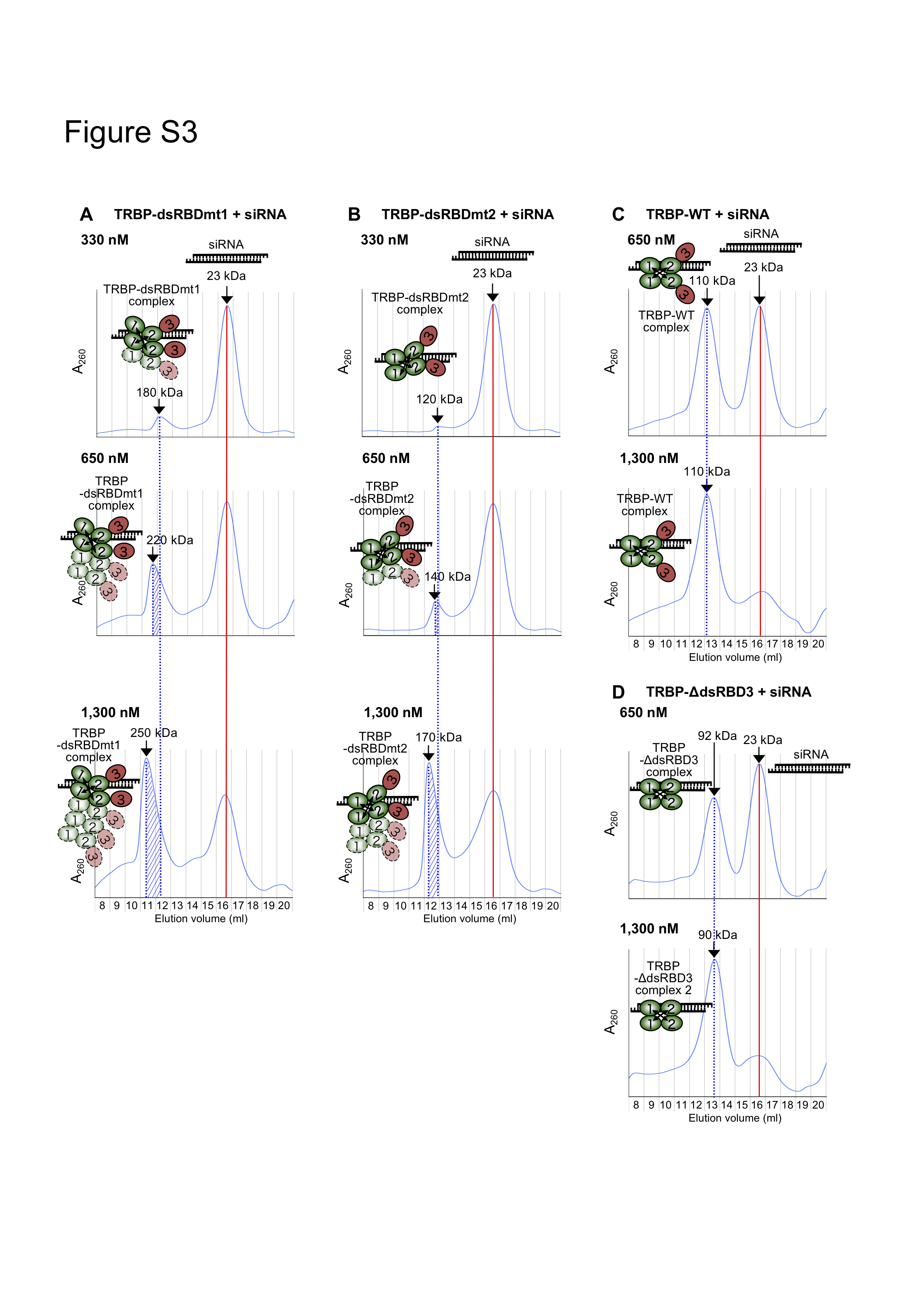

Supplement: Figure S3 — Gel filtration chromatography of purified TRBP-dsRBDmt1, TRBP-dsRBDmt2, TRBP-WT, and TRBP-ΔdsRBD3 proteins with siRNA. Gel filtration chromatography patterns of non-labeled siLuc-36 (300 nM) with increasing amounts of TRBP-dsRBDmt1 (A) and TRBP-dsRBDmt2 (B) (330, 650, 1,300 nM), TRBP-WT (C) and TRBP-ΔdsRBD3 (D) (650, 1,300 nM). Red lines indicate the positions of siRNA peaks. Blue dotted lines, the peaks of TRBP-dsRBDmt1 (A), TRBP-dsRBDmt2 (B), TRBP-WT (C), and TRBP-ΔdsRBD3 (D) with 330 nM siRNA. The shifted areas of these peaks in A and B were represented as blue dashed lines. Note that the peaks in C and D were not shifted even when these protein concentrations increased. (TIFF) [file pone.0063434.s003.tiff]

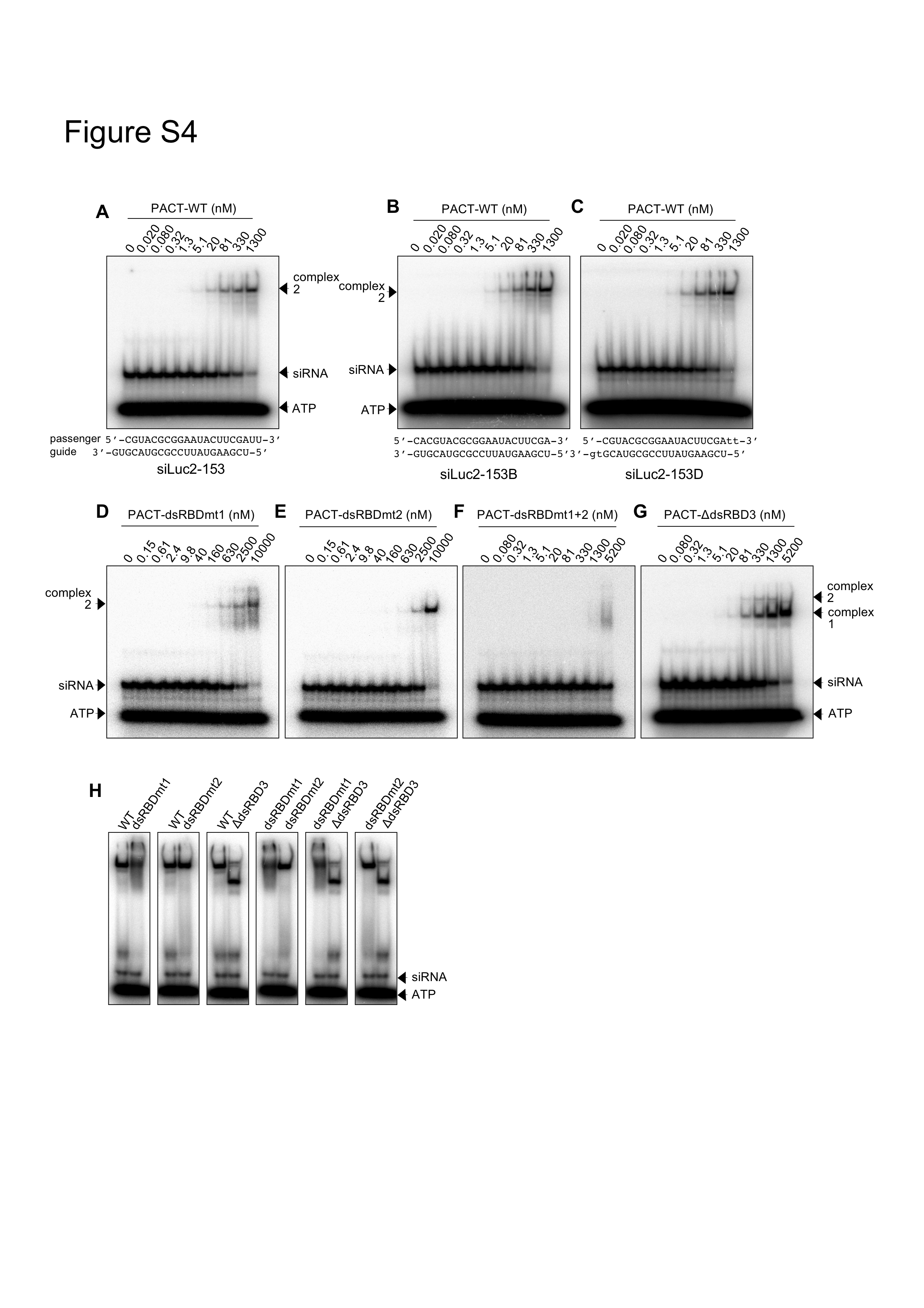

Supplement: Figure S4 — EMSA patterns of PACT-WT and its mutant proteins. (A–G) EMSA patterns of PACT-WT with 32P-labeled siLuc2-153 (A), siLuc2-153B (B), and siLuc2-153D (C), and those of PACT-dsRBDmt1 (D), PACT-dsRBDmt2 (E), PACT-dsRBDmt1+2 (F), and PACT-ΔdsRBD3 (G) with 32P-labeled siLuc2-153. 32P-labeled siRNA (0.50 nM) was incubated with increasing amounts of PACT-WT and its mutant proteins, as indicated. Lower cases in C in siLuc2-153D sequence indicate DNAs. Arrows indicate positions of the migrating complexes 1 and 2, in addition to siRNA and ATP. (H) Comparison of siRNA complex mobilities of PACT-WT, PACT-dsRBDmt1, PACT-dsRBDmt2, PACT-dsRBDmt1+2 and PACT-ΔdsRBD3 protein (1,300 nM). (TIFF) [file pone.0063434.s004.tiff]

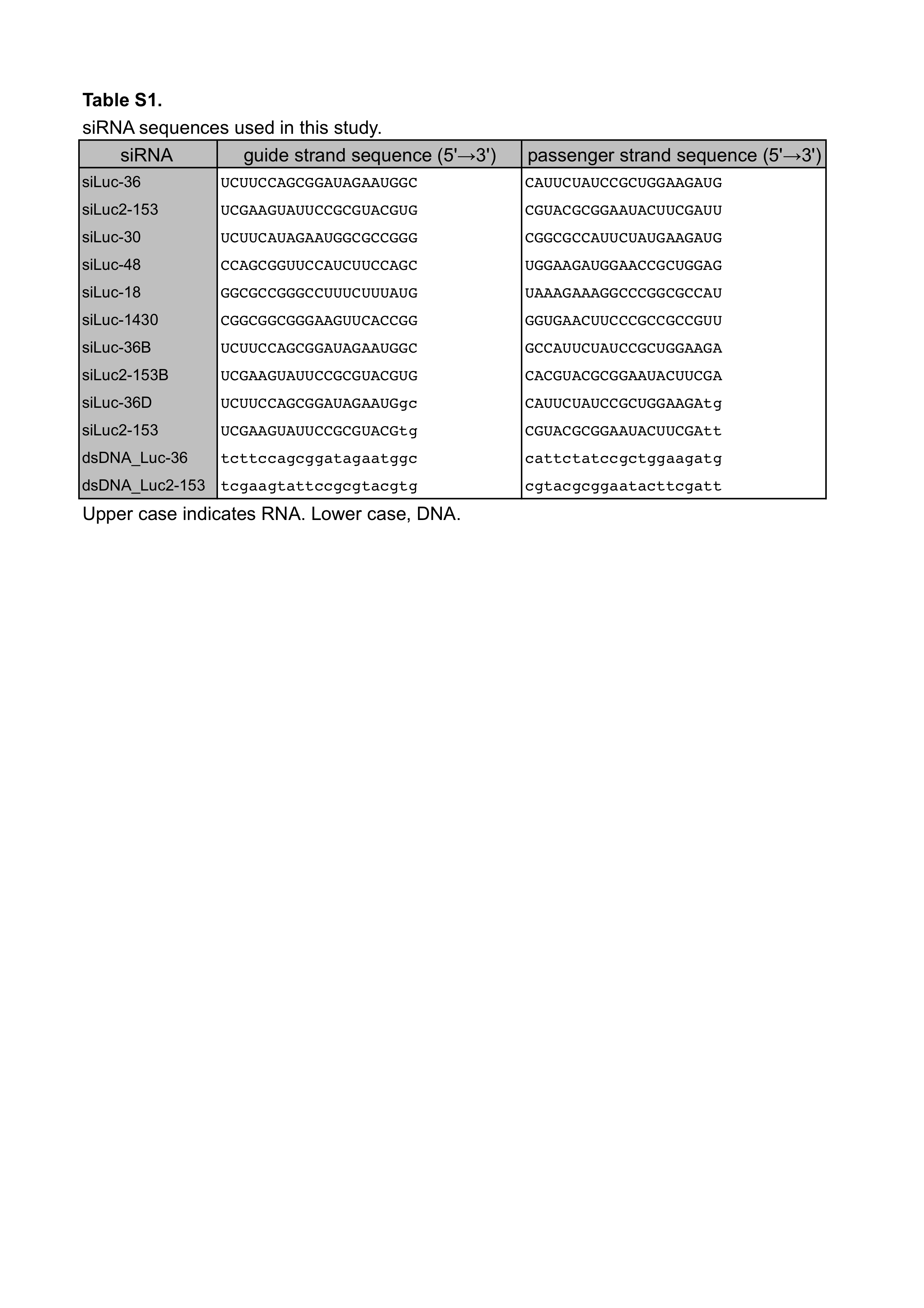

Supplement: Table S1 — siRNA sequences used in this study. (TIFF) [file pone.0063434.s005.tiff]

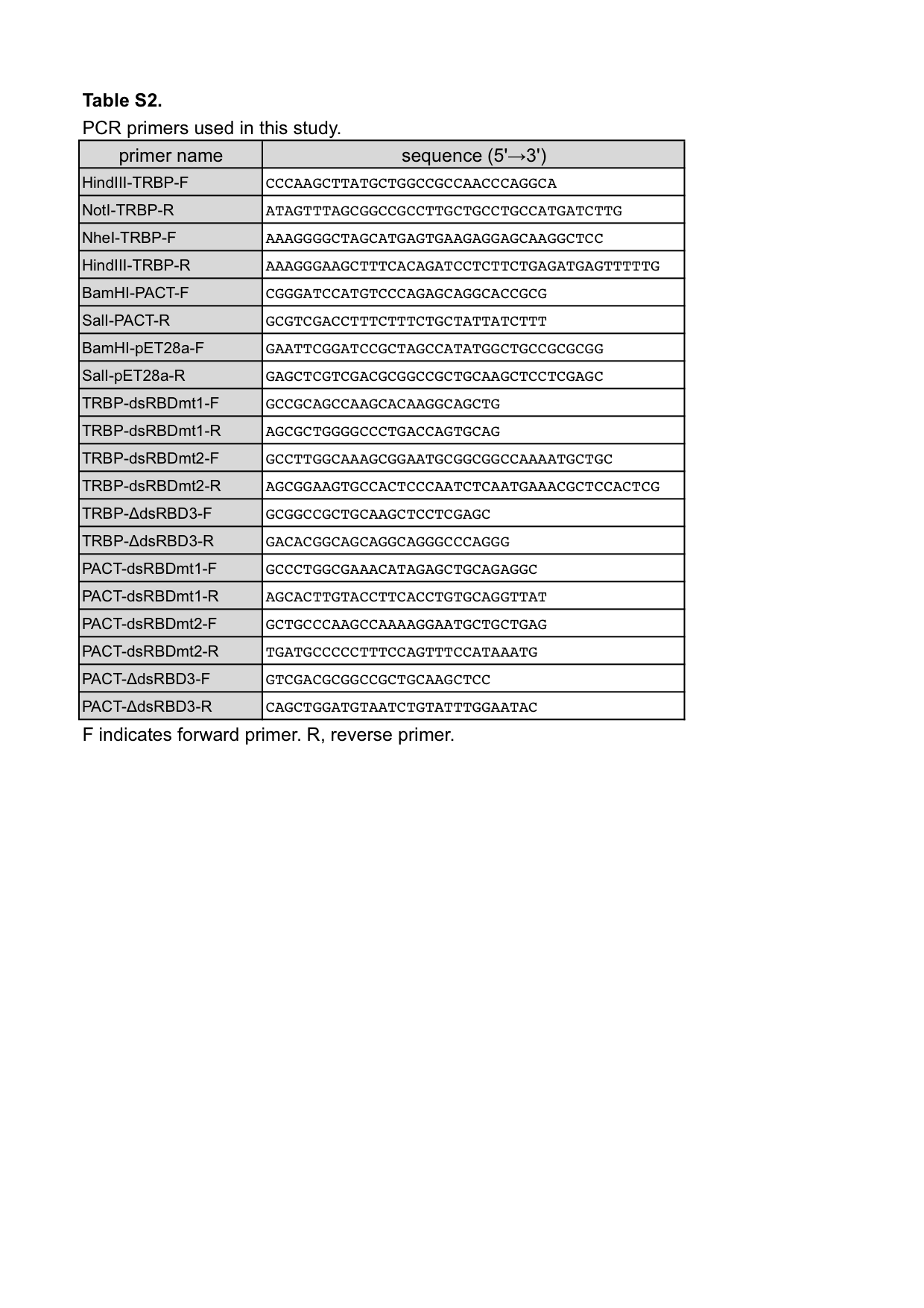

Supplement: Table S2 — PCR primers used in this study. (TIFF) [file pone.0063434.s006.tiff]
